# Efficient Capsid Antigen Presentation From Adeno-Associated Virus Empty Virions *In Vivo*

**DOI:** 10.3389/fimmu.2018.00844

**Published:** 2018-04-19

**Authors:** Xiaolei Pei, Lauriel Freya Earley, Yi He, Xiaojing Chen, Nikita Elexa Hall, Richard Jude Samulski, Chengwen Li

**Affiliations:** ^1^Chinese Academy of Medical Sciences and Peking Union Medical College, Institute of Hematology and Blood Diseases Hospital, Tianjin, China; ^2^Gene Therapy Center, University of North Carolina at Chapel Hill, Chapel Hill, NC, United States; ^3^Department of Pharmacology, University of North Carolina at Chapel Hill, Chapel Hill, NC, United States; ^4^Department of Pediatrics, University of North Carolina at Chapel Hill, Chapel Hill, NC, United States

**Keywords:** adeno-associated virus, capsid, antigen presentation, CD8+ T cells, empty virions

## Abstract

Adeno-associated virus (AAV) vectors have been successfully applied in clinical trials for hemophilic patients. Although promising, the clinical results suggest that the capsid-specific CD8+T cell response has a negative effect on therapeutic success. In an *in vitro* analysis using an engineered AAV virus carrying immune-dominant SIINFEKL peptide in the capsid backbone, we have previously demonstrated that capsid antigen presentation from full (genome containing) AAV capsids requires endosome escape and is proteasome dependent and that no capsid antigen presentation is induced from empty virions. In the present study, we examined capsid antigen presentation from administration of empty virions in animal models. In wild-type mice, similar to AAV full particles, capsid antigen presentation from AAV empty virion infection was dose dependent, and the kinetics studies showed that antigen presentation was detected from 2 to 40 days after AAV empty virion administration. In the transporter associated with antigen processing 1 deficient (TAP−/−) mice, capsid antigen presentation was inhibited from both AAV full and empty virions, but higher inhibition was achieved from AAV full particle administration than that from empty virions. This indicates that the pathway of capsid antigen presentation from AAV transduction is dependent on proteasome-mediated degradation of AAV capsids (mainly for full particles) and that the endosomal pathway may also play a role in antigen presentation from empty particles but not full virions. The capsid antigen presentation efficiency from AAV preparations was positively correlated with the amount of empty virions contaminated with full particles. Collectively, the results indicate that contamination of AAV empty virions induces efficient antigen presentation *in vivo* and the mechanism of capsid antigen presentation from empty virions involves both endosomal and proteasomal pathways. The elucidation of capsid antigen presentation from AAV empty virions may allow us to rationally design effective strategies to prevent elimination of AAV transduced target cells by capsid specific CD8+ T cells.

## Introduction

Adeno-associated virus (AAV) vectors have been successfully used to transduce hepatocytes in Phase I clinical trials in patients with hemophilia B (1–4). However, clinical results have suggested that capsid specific cytotoxic T lymphocytes (CTLs) eliminated AAV-transduced hepatocytes, which resulted in therapeutic failure (1–4). These observations pose an outstanding concern regarding capsid antigen presentation in AAV-transduced cells, which are recognized and eliminated by capsid specific CTLs in clinical trials. Most peptides loaded on MHC class I molecules are generated by proteasome degradation of newly synthesized ubiquitinated proteins, but exogenous proteins can also be presented on MHC class I molecules through cross-presentation. Two main intracellular pathways for cross-presentation have been described: endocytic (TAP and proteasome-independent) and cytosolic (TAP and proteasome-dependent) MHC class I peptide loading (5). In the TAP-independent pathway, exogenous antigens that have been endocytosed are degraded by proteases, and the resulting peptides bind to MHC class I molecules in late endosomes and lysosomes. In the TAP-dependent pathway, the internalized exogenous antigens are transferred from the endocytic pathway to the cytosol and degraded by proteasomes; the resulting peptides are then transported to the ER *via* TAP. Using pharmacological agents and AAV mutants, we have demonstrated that the classic MHC class I antigen presentation pathway plays a major role in AAV capsid antigen cross-presentation in AAV-transduced cells *in vitro* (6). However, the mechanism of capsid antigen cross-presentation from AAV-transduced cells *in vivo* is perhaps different from that *in vitro* due to the far more complex environment.

Adeno-associated virus vectors purified from cesium chloride (CsCl) density gradients have been applied in clinical trials; however, this purification approach is not scalable. Recently, ion-exchange chromatography has been studied to purify AAV vectors (7–10). Unlike the CsCl approach, the chromatographic method cannot separate genome-containing particles of AAV vectors (full particles) from empty virions as it relies on the charge of the capsid surface. The contamination of vector preparations by empty virions may inhibit transduction of genome containing AAV vectors and potentially increases the virus capsid antigen load in transduced cells as dose is determined by genome copy number, not by total number of capsids (10). Although empty AAV particles contain the identical protein components required for trafficking as that in full particles, our preliminary results demonstrated that capsid antigen presentation was significantly reduced in AAV transduced cells infected with AAV2 empty virions compared to full particles *in vitro* (6). This phenomenon may be interpreted as insufficient escape of these empty virions from the endosome (11). Recently, empty AAV capsids were proposed to function as decoys in clinical trials to allow AAV full particles to escape neutralizing antibodies (12). However, liver damage was observed following systemic administration of AAV preparations contaminated with empty particles (13), which alludes to the possibility that the contamination of empty particles leads to increased capsid antigen presentation due to an increase in the number of capsids infused into the patients. In this study, our *in vivo* results demonstrate that capsid antigen presentation was induced from AAV empty virions. The capsid antigen presentation from empty and full AAV particles was dose dependent and occurred at an early time period after administration for AAV empty particles when compared to full particles. Contrary to AAV full particles, the capsid antigen presentation from empty virions may involve both antigen presentation pathways: endosomal dependent and proteasomal dependent. More efficient antigen presentation was observed when a high amount of empty virions were included in AAV preparations.

## Materials and Methods

### Cells and Virus

HEK293 cells were maintained at 37°C and 5% CO_2_ in Dulbecco’s modified Eagle’s medium with supplementation of 10% heat-inactivated fetal calf serum, 100 U/ml penicillin, and 100 g/ml streptomycin. AAV full particle virus production was previously described using three plasmid cotransfection in 293 cells (14). AAV empty virions were produced by transfection of 293 cells with the AAV-OVA helper and Ad helper plasmids without AAV transgene cassette plasmid and then purified by CsCl gradient centrifugation. The titer of the produced empty particles was determined using a Western blot with antibodies specifically detecting intact virions (A20 and ADK8 antibodies for AAV2 and AAV8, respectively). The full particle virus titer was determined by Southern dot blot. The denatured empty virions were verified by Western blot with A20 or B1 antibody. 293/H-2Kb cells are a derivative of the 293 cell line stably expressing mouse MHC class I (H-2Kb).

### Mice

C57BL/6 mice, TAP−/− mice which are unable to present cytosolic antigens to class I-restricted cytotoxic T cells by delivery of peptides across the endoplasmic reticulum membrane to class I molecules, and OT-1/Rag-1 mice with expression of an H2Kb-restricted T-cell receptor specific for the OVA-derived SIINFEKL peptide were purchased from Jackson Laboratory (Bar Harbor, ME, USA). All mice were maintained in a specific pathogen-free facility at the University of North Carolina at Chapel Hill. The University of North Carolina Institutional Animal Care and Use Committee approved all procedures.

### Enzyme-Linked Immunosorbent Assay (ELISA) for Human Alpha-1-Antitrypsin (AAT)

Alpha-1-antitrypsin concentration in blood was carried out using an ELISA as described previously (15).

### *In Vivo* T Cell Proliferation

AAVOVA vector was injected in C57BL/6 mice or TAP−/− mice *via* retro-orbital administration. Spleen cells were collected from OT-1 mice and labeled with CFSE, then transfused into C57BL/6 mice or TAP−/− mice after AAV administration. At indicated time following OT-1 T cell transfer, spleen cells were harvested, and the frequency of T cells in proliferation status was determined by flow cytometer (16). Consistent with our previous study, majority of OT-1 spleen cells in proliferation were CD8+ T cells *in vitro* and *in vivo* after AAV/OVA capsids were applied (Figure S1 in Supplementary Material). No significant CD4+ cell proliferation was observed in these studies, so we chose to perform proliferation analysis using whole spleen cells.

### Statistical Analysis

The Student’s *t*-test was used to perform statistical analysis. *P* < 0.05 was considered a statistically significant difference.

## Results

### AAV2 Empty Virions Induce Efficient Capsid Antigen Presentation *In Vivo*

**Figure 1:**
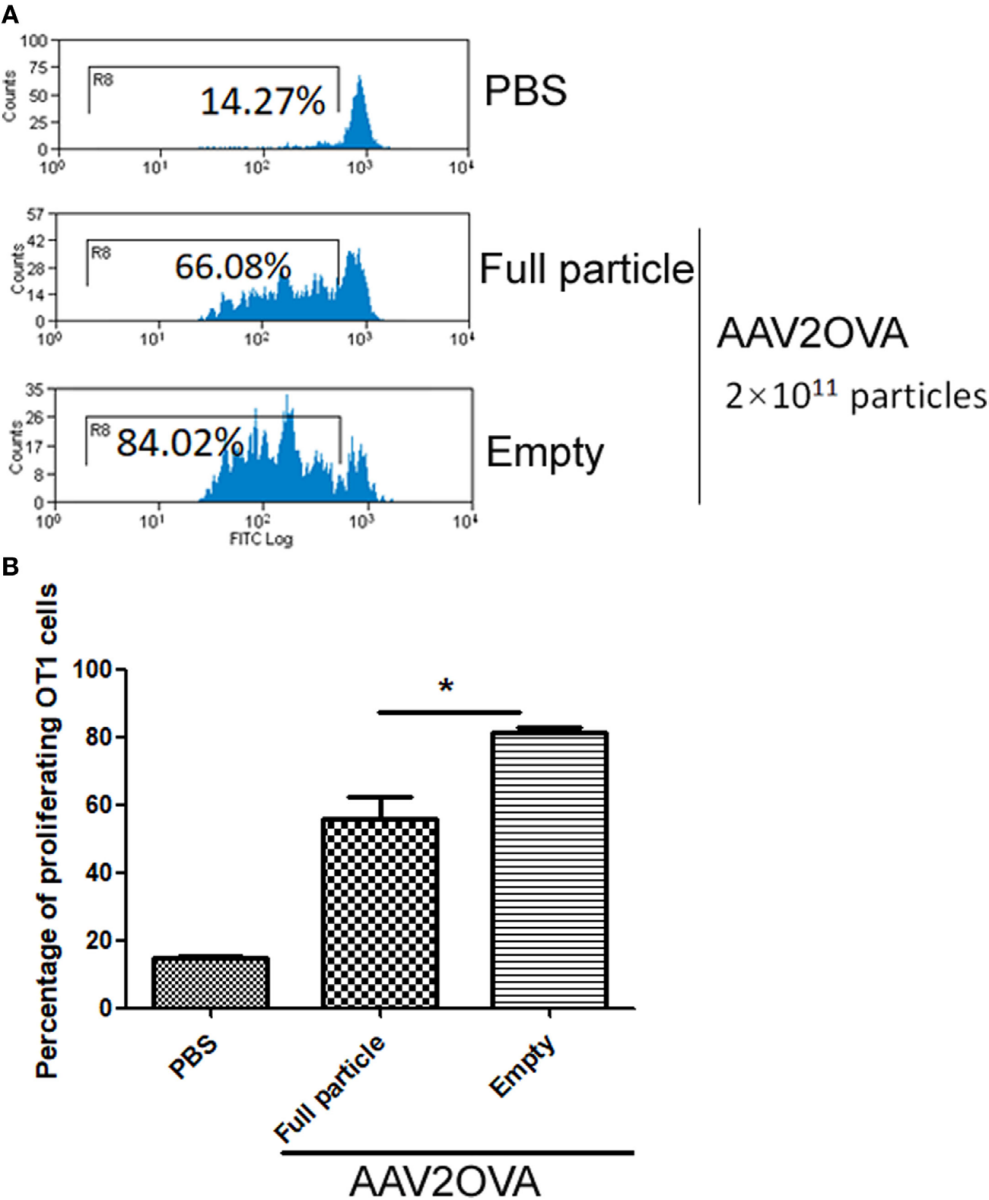
Efficient capsid antigen presentation from systemic administration of AAV2OVA empty virions in mice. 2 × 10^11^ particles of AAV2OVA empty virions or full particle vectors encoding transgene alpha-1-antitrypsin were intravenously injected into C57BL/6 mice *via* retro-orbital vein. At day 3 post adeno-associated virus (AAV) injection, 5 × 10^6^ CFSE-labeled OT-1 T cells were transferred into the mice. Ten days after transfer of OT-1 cells, the proliferation of OT-1 T cells was measured by flow cytometry. **(A)** Representative flow cytometric histograms. **(B)** Average T cell proliferation and SD for four mice. *p < 0.05 compared to mice with AAV full particle treatment.

In our previous studies, we used an engineered AAV2 capsid by swapping the immune-domain epitope, SIINFEKL, from ovalbumin protein into the AAV virion HI loop and demonstrated that capsid antigen presentation is elicited from AAV2-transduced liver cells in mice (17). In reality, empty particles are impossible to completely remove from the AAV virus preparation, especially in AAV virion purification using column affinity. This raises a concern as to whether the contamination of empty virions in AAV preparations increases capsid antigen presentation in transduced cells. To evaluate this, we injected 2 × 10^11^ particles of AAV2OVA full capsids or empty virions into C57BL/6 mice *via* systemic administration and 3 days later, CFSE conjugated OT-1 spleen cells were infused into the mice. At day 10 post OT-1 cell transfusion, the mice were sacrificed, and spleen cells were collected for OT-1 spleen cell proliferation analysis. Surprisingly, 80% of OT-1 cells were in division status in mice receiving AAV2OVA empty virions, a slightly higher value than that observed in mice with AAV2OVA full particle application (Figure 1, *p* < 0.05). This result indicates that empty virions also induce capsid antigen presentation *in vivo*.

### Similar Kinetics of Capsid Antigen Presentation Between Full Particles and Empty Virions

**Figure 2:**
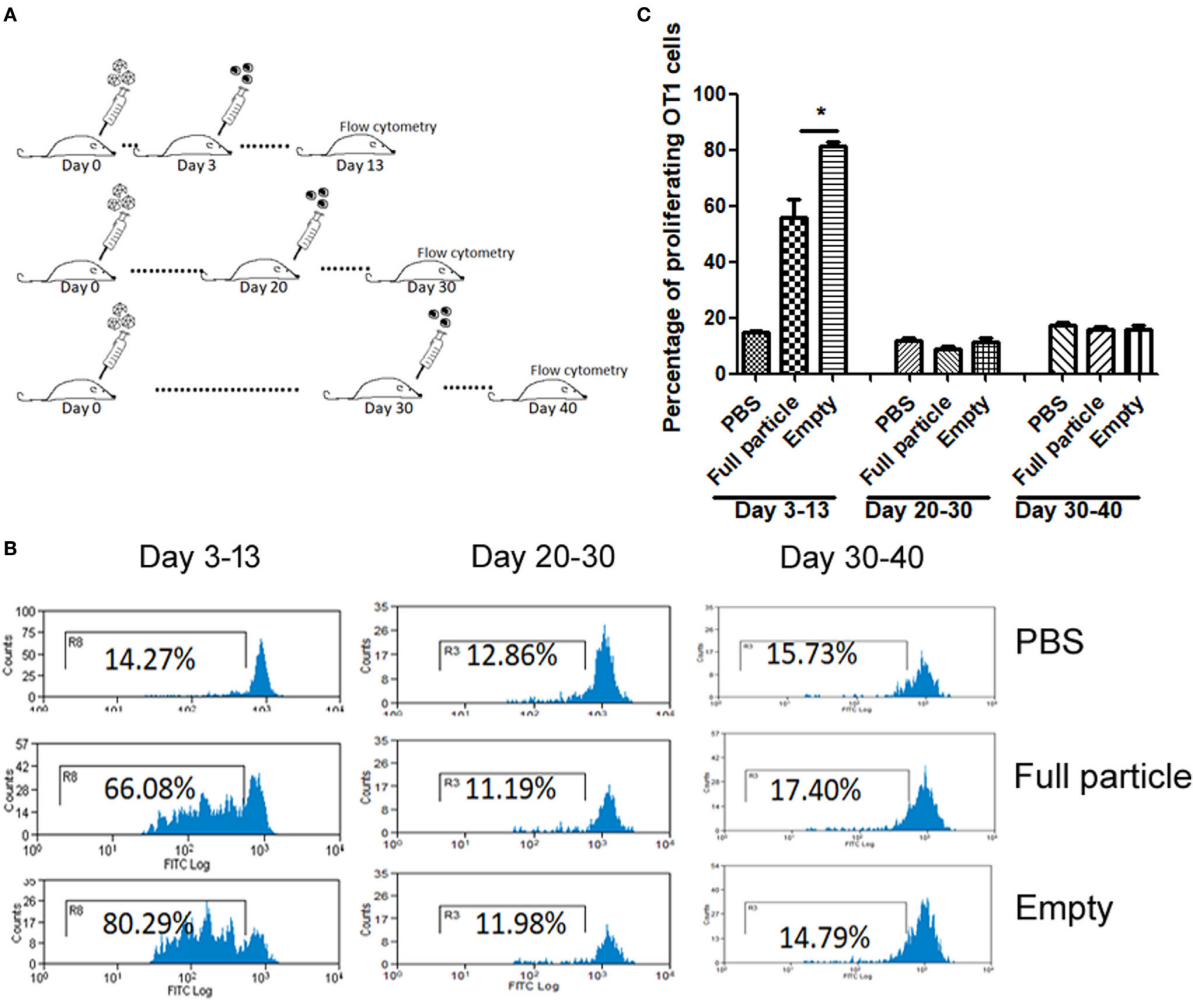
The kinetics of capsid antigen presentation from AAV2OVA empty virions in mice. AAVOVA viruses (empty or full) at a dose of 2 × 10^11^ were systemically administered *via* retro-orbital injection into C57BL/6 mice, and at the indicated time points, CFSE-labeled OT-1 T cells were transfused. At day 10 post OT-1 infusion, the proliferation of OT-1 T cells was analyzed by flow cytometry. **(A)** A scheme for time points of adeno-associated virus administration and OT-1 cells injection as well as assay for OT-1 spleen cell proliferation. **(B)** Representative flow cytometric histograms. **(C)** Average T cell proliferation and SD for four mice.

**Figure 3:**
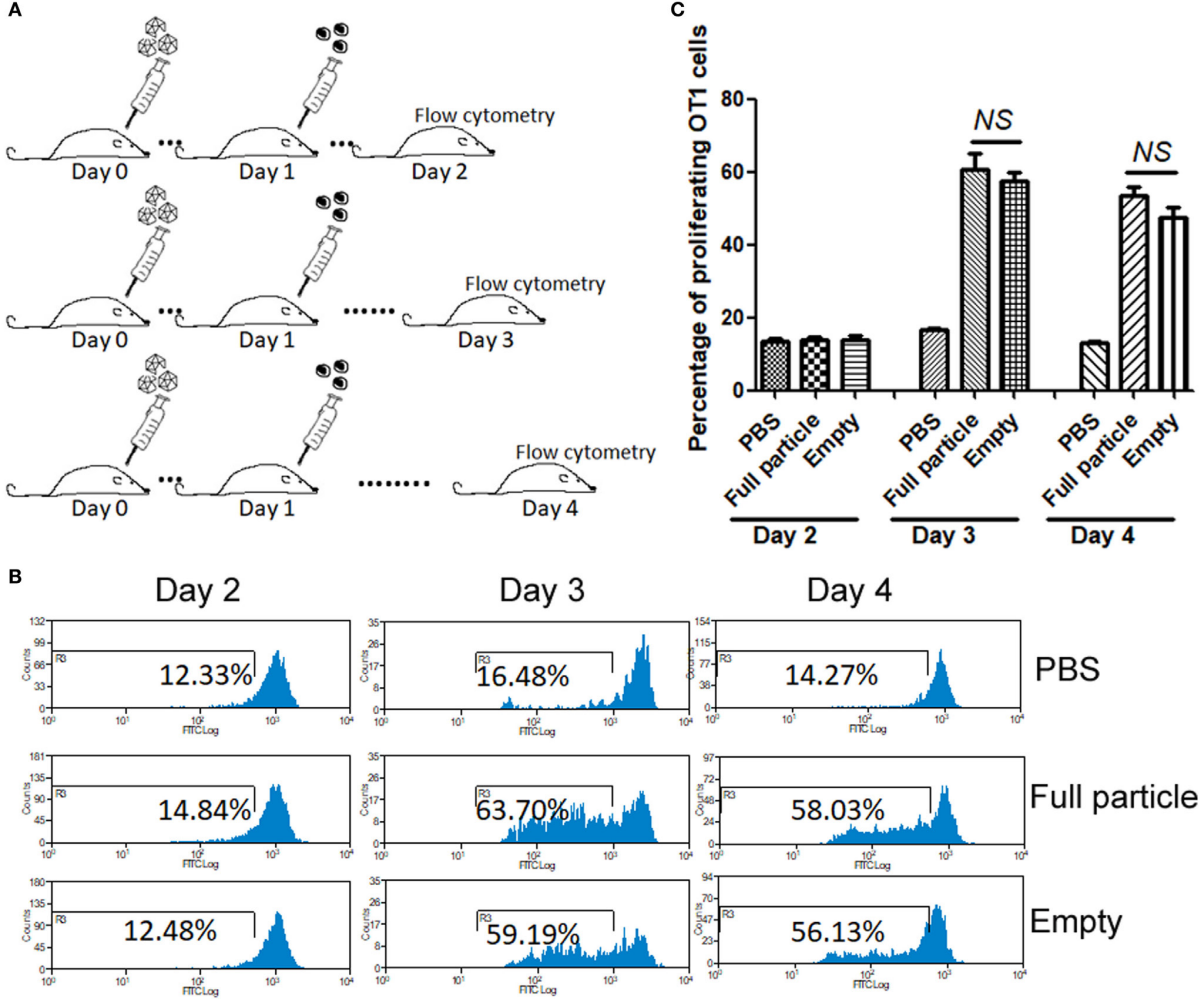
Capsid antigen presentation from AAV2OVA empty virions at early time points after administration in mice. 2 × 10^11^ particles of AAVOVA viruses were systemically injected *via* retro-orbital vein into C57BL/6 mice. One day later, CFSE-labeled OT-1 spleen cells were transferred into the mice. At days 1, 2, and 3 post OT-1 cell infusion, the proliferation of OT-1 cells was analyzed by flow cytometry. **(A)** A scheme for time points of adeno-associated virus administration and OT-1 cells injection as well as assay for OT-1 spleen cell proliferation. **(B)** Representative flow cytometric histograms. **(C)** Average T cell proliferation and SD for four mice.

Our previous study has demonstrated that the efficiency of capsid antigen presentation from full particle transduction gradually decreases over time, and higher capsid antigen presentation occurs at an early time after AAV transduction *in vivo* (18). Next, we examined whether capsid antigen presentation from empty virion infection followed a similar trend to that of full particles. We injected 2 × 10^11^ particles of AAV2OVA empty virions into C57BL/6 mice, and at days 3, 20, and 30 post AAV administration, CFSE-conjugated OT-1 spleen cells were transfused. Ten days later post OT-1 injection, the efficiency of antigen presentation was analyzed. As shown in Figure 2, capsid antigen presentation was much lower when OT-1 cells were infused at days 20 and 30 than that when OT-1 cells were infused at day 3 post AAV empty virion application. No antigen presentation was observed at days 30 and 40 with OT-1 injection. These results indicate a similar kinetics of capsid antigen presentation between full particles and empty particle. In our previous study, we demonstrated that no capsid antigen presentation was observed in 293/H-2Kb cells infected with AAV2OVA empty virions *in vitro* (6). This prior experiment was performed within a short time of both AAV transduction (<48 h) and of incubation of AAV transduced cells with OT-1 spleens (<24 h), which is different from the latest experiment carried out with a relative long time of transduction and of interaction of AAV transduced cells with OT-1 spleen cells *in vivo*. To investigate the capsid antigen presentation efficiency from empty virions immediately followed by AAV infection *in vivo*, OT-1 spleen cells were infused at day 1 after AAV systemic administration, and antigen presentation was assayed at days 1, 2, and 3 post OT-1 cell injection. No capsid antigen presentation was detected at day 1 post OT-1 infusion, similar to the *in vitro* finding for capsid antigen presentation from empty virions, but contrary to that from full particles. Comparable capsid antigen presentation was demonstrated in days 2 and 3 post OT-1 cell transfer in mice receiving either full particles or empty virions (Figure 3). These results suggest that capsid antigen presentation occurs at an early phase after virion infection.

### Capsid Antigen Presentation From AAV2 Empty Virions Is Dose Dependent *In Vivo*

**Figure 4:**
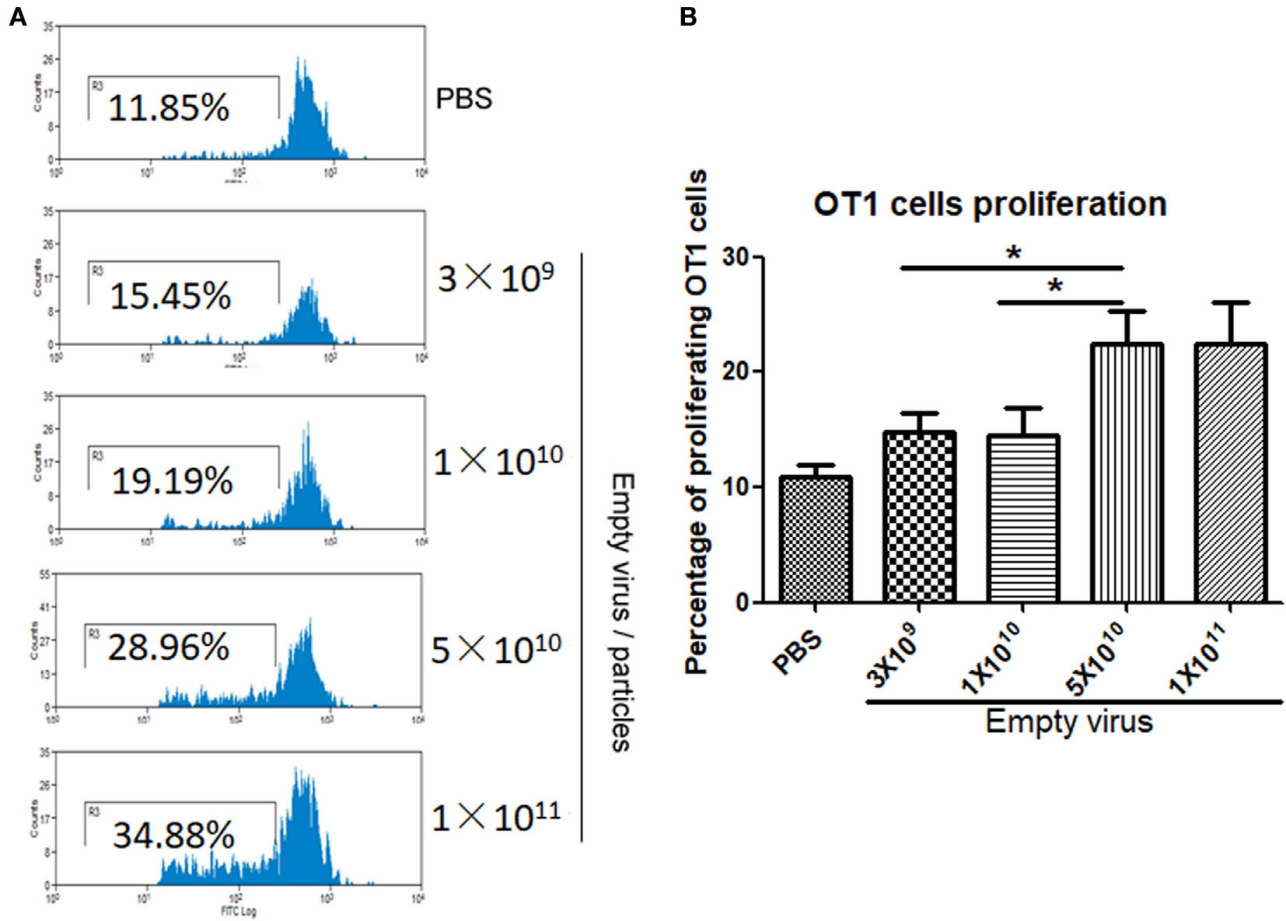
Capsid antigen presentation from AAV2OVA empty virions is dose responsive *in vivo*. Different doses of AAV2OVA empty vectors were intravenously injected into C57BL/6 mice and 3 days later, and CFSE-labeled OT-1 T cells were transferred into the mice. At day 10 post injection of OT-1 cells, T cell proliferation in the spleen was analyzed *via* flow cytometry. **(A)** Representative flow cytometric histograms. **(B)** Average T cell proliferation and SD of five mice. *p < 0.05 when compared to mice with the low dose of AAV treatment.

To study dose-dependent capsid antigen presentation, we injected mice with different doses of AAV2OVA empty virions and infused OT-1 spleen cells 3 days later. Capsid antigen presentation was analyzed at day 10 post OT-1 infusion. As shown in Figure 4, when 1 × 10^10^ empty virions were administered, we did not detect any capsid antigen presentation. However, when 5 × 10^10^ empty virions were injected, capsid antigen presentation was observed. This result is consistent with the observation from capsid antigen presentation using AAV2 full particles in our previous report, in which antigen presentation is dose dependent (18).

### Capsid Antigen Presentation From AAV8 Empty Virions *In Vivo*

**Figure 5:**
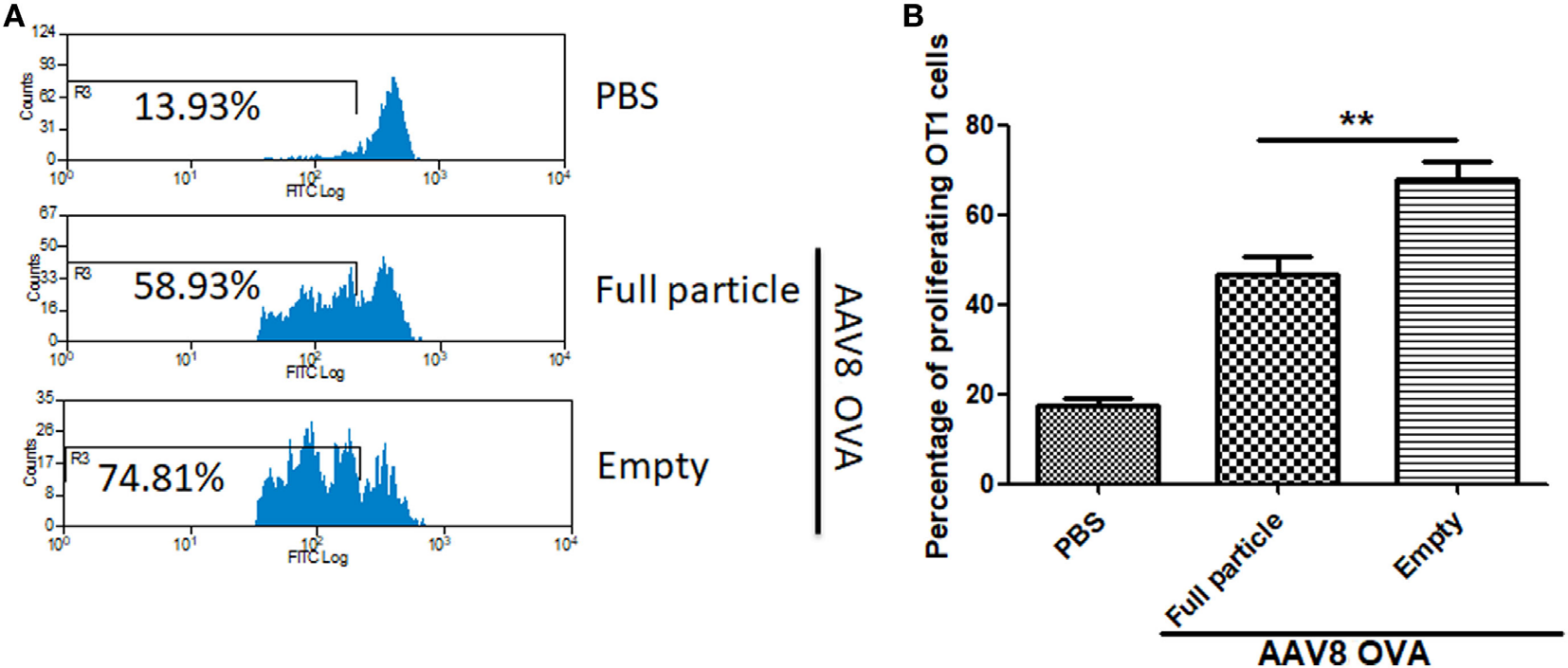
The capsid antigen presentation from AAV8OVA empty virions in mice. 2 × 10^11^ particles of AAV8OVA viruses (empty or full) were injected into C57BL/6 mice *via* retro-orbital vein, and at day 3 post AAV8OVA injection, 5 × 10^6^ CFSE-labeled OT-1 T cells were transferred. Ten days posttransfer, proliferation of OT-1 cells was measured by flow cytometry. **(A)** Representative flow cytometric histograms. **(B)** Average T cell proliferation and SD for four mice. *p < 0.05 when compared to mice treated with AAV8OVA full particles.

To study whether capsid antigen presentation from empty virions also applied to other serotypes, we made AAV8OVA empty virions. After injection of 2 × 10^11^ AAV8OVA empty particles, OT-1 spleen cells were transfused at day 3 and 10 days later, the mice were sacrificed, and spleen cells were collected for an OT-1 spleen cell proliferation assay. As shown in Figure 5, similar to capsid antigen presentation from AAV2 empty virions, efficient capsid antigen presentation from AAV8 empty virions was also detected and was higher than that from AAV8 full particles. These data implicate that capsid antigen presentation from empty virions can likely be induced from any AAV serotype *in vivo*.

### Contamination of Empty Virions in AAV Preparation Increases Capsid Antigen Presentation

**Figure 6:**
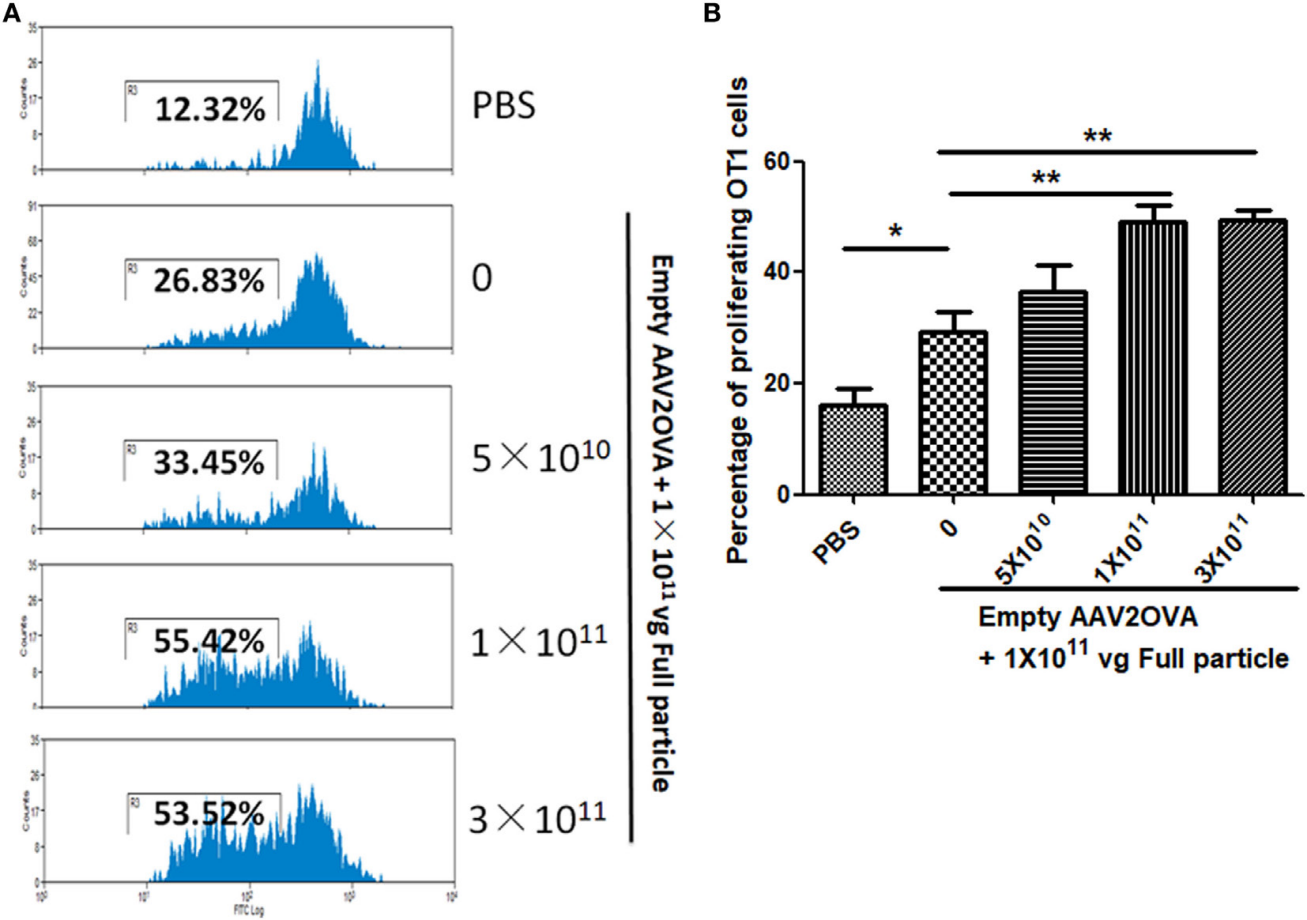
Contamination of empty virions in adeno-associated virus (AAV) preparation increases capsid antigen presentation. Different amounts of AAV2OVA empty virions were mixed with 1 × 10^11^ particles of AAV2OVA/alpha-1-antitrypsin (AAT) vectors and then administered into C57BL mice *via* retro-orbital injection. At day 3 post AAV injection, OT-1 spleen cells were transfused. Ten days later, mice were killed, and spleen cells were collected for OT-1 cell proliferation assay. **(A)** Representative flow cytometric histograms. **(B)** Average T cell proliferation and SD from five mice.

**Figure 7:**
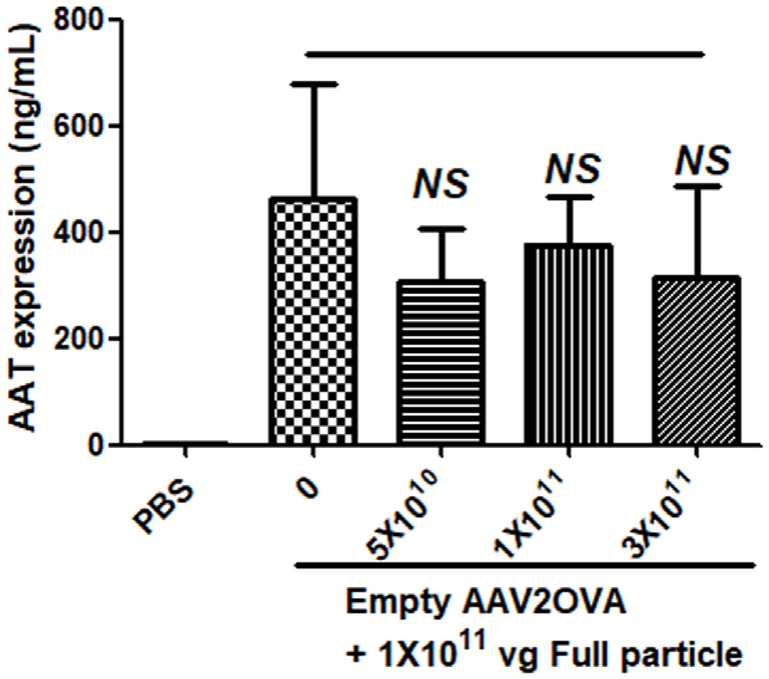
No effect of empty virions on transgene expression from adeno-associated virus full particles after systemic administration. Blood was collected from mice in Figure 6 when mice were sacrificed for capsid antigen presentation analysis. Transgene alpha-1-antitrypsin (AAT) expression was detected by enzyme-linked immunosorbent assay. The result is represented as the average and SD of five mice.

To study the contribution of empty virions in AAV preparation to capsid antigen presentation, we mixed different amounts of empty virions into 1 × 10^11^ particles of AAV full particles of AAV2OVA capsids containing a CBA-driven AAT genome. At day 3 after injection of the mixture of AAV full particles with empty virions into mice, OT-1 spleen cells were infused and 10 days later the spleen cells were harvested for antigen presentation analysis. As described earlier, capsid antigen presentation was detected when 1 × 10^11^ particles of full particles only were injected, and significantly increased when empty virions were added (Figure 6). This study supports the hypothesis that the contamination of empty virions in AAV preparation increases AAV antigen load, which may induce higher elimination of AAV-transduced target cells mediated by capsid-specific CTLs. To examine whether the contamination of empty virions had an impact on AAV transduction from full particles, blood was collected at day 13 post AAV injection when mice were sacrificed for capsid antigen presentation analysis, and transgene AAT expression was measured by ELISA. As shown in Figure 7, the AAT level in blood was similar among the different groups regardless of the amount of empty virions added to the fixed dose of AAV2 full particles.

### Blockage of Proteasome Pathway Partially Inhibits Capsid Antigen Presentation From Empty Capsids

**Figure 8:**
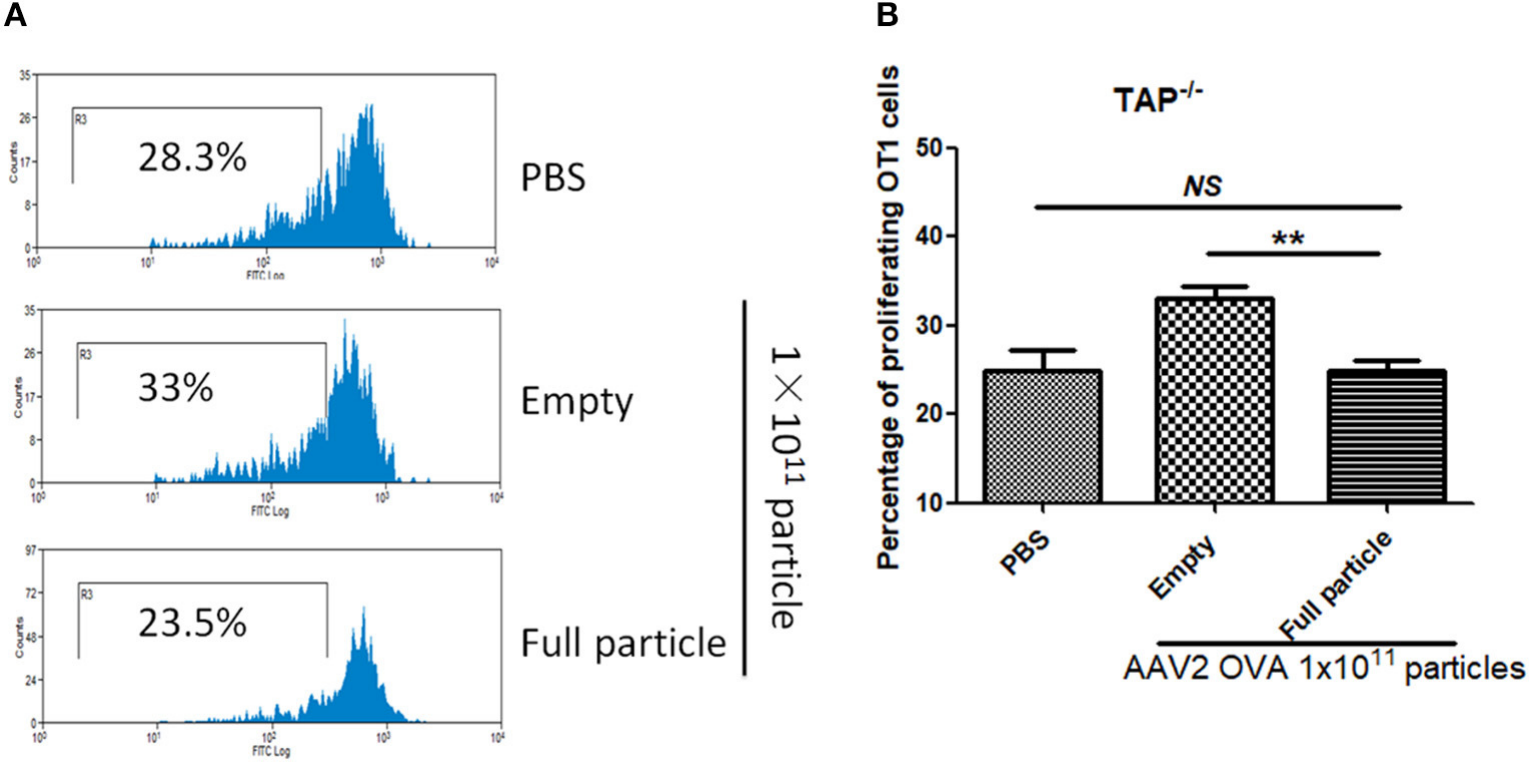
Blockage of proteasome pathway inhibits capsid antigen presentation from empty capsid. 1 × 10^11^ particles of AAV2OVA vectors (empty or full) were intravenously injected into TAP−/− mice *via* retro-orbital vein. At day 3, OT-1 cells were administered into the mice. Ten days later, spleen cells from mice were harvested for capsid antigen presentation analysis. **(A)** Representative flow cytometric histograms in TAP−/− mice treated with adeno-associated virus particles. **(B)** Average T cell proliferation and SD for five mice. **p < 0.01 when compared to mice treated with AAV2OVA full particles in TAP−/− mice.

In our previous study, we demonstrated that capsid antigen presentation from AAV-transduced cells is proteasome dependent and requires endosomal escape (6). Utilization of proteasome inhibitors completely blocks capsid antigen presentation in 293/H-2Kb cells in a short incubation period. To study which antigen presentation pathway is involved in capsid antigen presentation *in vivo* after AAV transduction, TAP−/− mice were chosen since TAP deficiency blocks antigen presentation from proteasome-mediated degradation. 1 × 10^11^ particles of AAV2OVA full particles were administered into TAP−/− mice and wild-type C57BL/6 mice. At day 3 post AAV injection, OT-1 cells were transfused into the mice and 10 days later, spleen cells were collected for a capsid antigen presentation assay. Consistent to the finding in 293/H-2Kb cells, no capsid antigen presentation was documented in TAP−/− mice, although significantly higher capsid antigen presentation was observed in wild-type mice receiving AAV2OVA full particles when compared to control mice only receiving PBS injection (Figure 8). When 1 × 10^11^ particles of AAV2OVA virions were administered in TAP−/− mice, there was no significant difference in OT1 cell proliferation between mice injected with PBS or full particles; however, capsid antigen presentation from empty capsids was higher than that of AAV2OVA full particles. This result suggests that the capsid antigen presentation from AAV empty virions may use different mechanisms to process antigens, and this process is less dependent on TAP antigen presentation. It was noted that OT-1 cell proliferation in TAP−/− control mice was higher than that in wild-type C57BL/6 mice, the difference may result from the different immune background of mouse strains.

## Discussion

In this study, we demonstrated that efficient capsid antigen presentation, as measured by CD8+ T-cell proliferation, was achieved from administration of AAV empty virions in wild-type mice. In agreement with the capsid antigen presentation from AAV full particles in mice, the capsid antigen presentation from AAV2 empty particles was dose dependent and detected with higher presentation than full particles at the early phase of AAV application. While the TAP pathway plays a major role in capsid antigen presentation from AAV full particles, both TAP and endosomal pathways may be involved in the capsid antigen presentation from empty virions *in vivo*. Contamination of empty virions in AAV preparation resulted in higher amounts of T-cell proliferation, indicating higher amounts of capsid antigen presentation.

Our previous studies have demonstrated that AAV full particles induce efficient capsid antigen presentation in mice and our mechanism study showed that capsid antigen presentation is proteasomal dependent in an *in vitro* well-defined cell line (6, 18); however, the capsid antigen presentation was not detected from empty virions *in vitro* (6). These results raise the question as to how capsid antigen presentation occurs from AAV empty virions *in vivo*. In considering this, the following factors may be important: (1) different environments between AAV transduced cells *in vivo* and *in vitro* including cell–cell interactions or cells with components around cells, (2) intracellular trafficking, and (3) the long-term interaction of AAV virions with cellular proteins. These factors likely help to explain the inconsistent results obtained from the *in vitro* assay, in which no detectable capsid antigen presentation was observed in cells infected with AAV empty virions at 48 h post infection, and the *in vivo* administration of empty virions which induced capsid antigen presentation similar to that of AAV full particles in mice. There was a trend that capsid antigen presentation was higher in mice treated with AAV full particles before day 4 of transduction than that in mice receiving empty virions. After day 4, the capsid antigen presentation was more prominent in mice treated with empty virions than that with full particles. These results indicate that (1) capsid antigen presentation from full particles may occur faster than that from empty virions *in vivo*, which suggests a different mechanism of capsid antigen presentation between AAV full particles and empty virions and (2) the *in vitro* assay was not performed at a sufficiently long enough time point to demonstrate antigen presentation from empty capsids.

Using pharmacological agents and AAV mutants in an *in vitro* analysis, we have previously demonstrated that capsid antigen presentation from AAV full particles requires efficient endosomal escape into the cytosol and capsid degradation by proteasomes, supporting the hypothesis that the proteasomal pathway is involved in capsid antigen presentation from AAV-transduced cells (6). In the present study, we compared the efficiency of capsid antigen presentation induced from full particles or empty virions in wild-type mice and mice with TAP deficiency, in which antigen presentation from proteasome-mediated degradation of antigen is blocked. Efficient capsid antigen presentation was detected in wild-type mice treated with either full particles or empty virions. When compared to control TAP−/− mice without AAV administration, no increased capsid antigen presentation was observed in TAP−/− mice treated with either full particles or empty virions, instead, we observed a reduction in capsid antigen presentation for the full particles. This result implicates that both AAV full and empty capsids are capable of inducing capsid antigen presentation via the cytosolic pathway. However, it should be noted that a little higher capsid antigen presentation was detected in TAP−/− mice treated with empty virions than that with full particles, suggesting that the endosomal pathway may also be involved in capsid antigen presentation from empty virions. These results, based on the kinetics of capsid antigen presentation and the mechanism studies of capsid antigen presentation from AAV full particles and empty virions *in vivo*, are supported by the findings that AAV full particles can escape from endosomes and traffic to the nucleus more efficiently than empty virions (19). Although both full and empty virions contain VP1, which harbors both the phospholipase A2 (PLA2) domain and nuclear localization signals (NLSs) required for efficient intracellular trafficking (11, 20, 21), empty particles are deficient in exposure of the N-terminal of VP1/2 of AAV virions when compared to full particles (22).

One interesting finding from the present study is that efficient capsid antigen presentation was achieved from denatured AAV virions. Although we did not perform a mechanism study, we presume that the endosomal pathway would be involved due to the fact that most denatured capsids should be VP3 subunits since VP3 accounts for 80% VP subunits and does not have the PLA2 domain and NLSs on its N-terminal (20, 21). This finding also implicates that elimination of denatured AAV capsid subunits in AAV preparation has the potential to decrease AAV capsid antigen load.

It has been reported that the contamination of empty virions inhibits the transduction from AAV full particles after muscular injection (10). In this study, we did not observe the inhibition effect of contaminated empty virions on AAV transduction from full capsids after systemic administration, similar to the observation from Mingozzi et al. (12). This difference can be attributed to variable administration routes and different targeted tissues. When AAV vectors were intramuscularly administered, the majority of AAV viruses accumulated around the injection site, and the ratio of AAV particles per muscle fiber was very high. Therefore, the high amount of empty virions competed with full particles to bind to the muscle surface and inhibited transduction from full particles. However, after systemic administration, AAV viruses circulated in the blood and were bound to cells in every tissue of the body. Although high doses of AAV vectors, including empty virions and full particles, are used for systemic injection, the AAV capsids per targeted cell would likely have been lower compared to muscle injection and the empty virions were not concentrated enough to compete with full particles for cell binding and intracellular trafficking, so there is no inhibition effect of empty virions on transgene expression from AAV full particles using IV injection. It is expected that transgene expression will be decreased when an extra high dose of empty virions is used.

In conclusion, our study demonstrates that efficient capsid antigen presentation can be induced from AAV empty capsids. The efficiency of the capsid antigen presentation from AAV empty virions is dose dependent and occurs in the early periods after administration of empty virions. The proteasome pathway plays a role in capsid antigen presentation from both full and empty particles, but the endosomal pathway may also be involved in capsid antigen presentation from empty virions. Contamination of empty virions increases capsid antigen presentation from AAV transduction. In summary, studies on the capsid antigen presentation from AAV empty virions *in vivo* will allow us to design effective approaches to prevent and block capsid-specific CTL-mediated elimination of AAV transduced target cells in future clinical trials.

## Ethics Statement

This study was carried out in accordance with the recommendations of “University Research Policies and Procedures, Institutional Animal Care and Use Committee at UNC”. The protocol was approved by the Institutional Animal Care and Use Committee at UNC.

## Author Contributions

XP completed the majority of this research including Figures 1–8 and participated in project design. YH completed the experiment including Figure 2, XC worked as the technician to provide experiment skill support, and RS and CL designed the project.

## Conflict of Interest Statement

RS is the founder and a shareholder at Asklepios BioPharmaceutical and Bamboo Therapeutics, Inc. He holds patents that have been licensed by UNC to Asklepios BioPharmaceutical, for which he receives royalties. He has consulted for Baxter Healthcare and has received payment for speaking.
