# Corrigendum: Efficient Capsid Antigen Presentation From Adeno-Associated Virus Empty Virions *In Vivo*

**DOI:** 10.3389/fimmu.2019.03076

**Published:** 2020-02-04

**Authors:** Xiaolei Pei, Lauriel Freya Earley, Yi He, Xiaojing Chen, Nikita Elexa Hall, Richard Jude Samulski, Chengwen Li

**Affiliations:** ^1^Chinese Academy of Medical Sciences and Peking Union Medical College, Institute of Hematology and Blood Diseases Hospital, Tianjin, China; ^2^Gene Therapy Center, University of North Carolina at Chapel Hill, Chapel Hill, NC, United States; ^3^Department of Pharmacology, University of North Carolina at Chapel Hill, Chapel Hill, NC, United States; ^4^Department of Pediatrics, University of North Carolina at Chapel Hill, Chapel Hill, NC, United States

**Keywords:** adeno-associated virus, capsid, antigen presentation, CD8+ T cells, empty virions

In the original article, there was a mistake in the legend for **Figure 5** as published. We used an incorrect description of AAV particles. The correct legend appears below.

“**Figure 5**. The capsid antigen presentation from AAV8OVA empty virions in mice. 2 × 10^11^ particles of AAV8OVA viruses (empty or full) were injected into C57BL/6 mice *via* retro-orbital vein, and at day 3 post AAV8OVA injection, 5 × 10^6^ CFSE-labeled OT-1 T cells were transferred. Ten days posttransfer, proliferation of OT-1 cells was measured by flow cytometry. **(A)** Representative flow cytometric histograms. **(B)** Average T cell proliferation and SD for four mice. ^*^p < 0.05 when compared to mice treated with AAV8OVA full particles.”

Additionally, there was a mistake in Figure 8 as published. In Figure 8B, the significant difference between empty particles and full particles should be shown and not PBS and empty particles. The corrected Figure 8 appears below.

**Figure 8:**
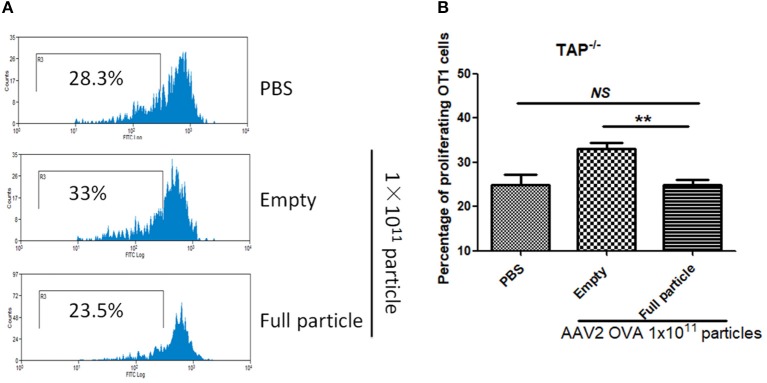
Blockage of proteasome pathway inhibits capsid antigen presentation from empty capsid. 1 × 10^11^ particles of AAV2OVA vectors (empty or full) were intravenously injected into TAP−/− mice *via* retro-orbital vein. At day 3, OT-1 cells were administered into the mice. Ten days later, spleen cells from mice were harvested for capsid antigen presentation analysis. **(A)** Representative flow cytometric histograms in TAP−/− mice treated with adeno-associated virus particles. **(B)** Average T cell proliferation and SD for five mice. **p < 0.01 when compared to mice treated with AAV2OVA full particles in TAP−/− mice.

Further, we erroneously indicated that “1.5 × 10^11^” instead of “1 × 10^11^” particles of AAV2OVA full particles were administered into TAP−/− mice and wild-type C57BL/6 mice.

A correction has been made to **Results** section, subsection **Blockage of Proteasome Pathway Partially Inhibits Capsid Antigen Presentation From Empty Capsids**, paragraph one:

“In our previous study, we demonstrated that capsid antigen presentation from AAV-transduced cells is proteasome dependent and requires endosomal escape (6). Utilization of proteasome inhibitors completely blocks capsid antigen presentation in 293/H-2Kb cells in a short incubation period. To study which antigen presentation pathway is involved in capsid antigen presentation *in vivo* after AAV transduction, TAP−/− mice were chosen since TAP deficiency blocks antigen presentation from proteasome-mediated degradation. 1 × 10^11^ particles of AAV2OVA full particles were administered into TAP−/− mice and wild-type C57BL/6 mice. At day 3 post AAV injection, OT-1 cells were transfused into the mice and 10 days later, spleen cells were collected for a capsid antigen presentation assay. Consistent to the finding in 293/H-2Kb cells, no capsid antigen presentation was documented in TAP−/− mice, although significantly higher capsid antigen presentation was observed in wild-type mice receiving AAV2OVA full particles when compared to control mice only receiving PBS injection (Figure 8). When 1 × 10^11^ particles of AAV2OVA virions were administered in TAP−/− mice, there was no significant difference in OT1 cell proliferation between mice injected with PBS or full particles; however, capsid antigen presentation from empty capsids was higher than that of AAV2OVA full particles. This result suggests that the capsid antigen presentation from AAV empty virions may use different mechanisms to process antigens, and this process is less dependent on TAP antigen presentation. It was noted that OT-1 cell proliferation in TAP−/− control mice was higher than that in wild-type C57BL/6 mice, the difference may result from the different immune background of mouse strains.”

The authors apologize for this error and state that this does not change the scientific conclusions of the article in any way. The original article has been updated.

