# Supplementary material for: Efficient Capsid Antigen Presentation From Adeno-Associated Virus Empty Virions In Vivo
Source: Front Immunol. 2018 Apr 19;9:844. doi: 10.3389/fimmu.2018.00844 (PMC5916967; doi:10.3389/fimmu.2018.00844)
Supplement: Supplementary file 1 [file presentation_1.PDF]

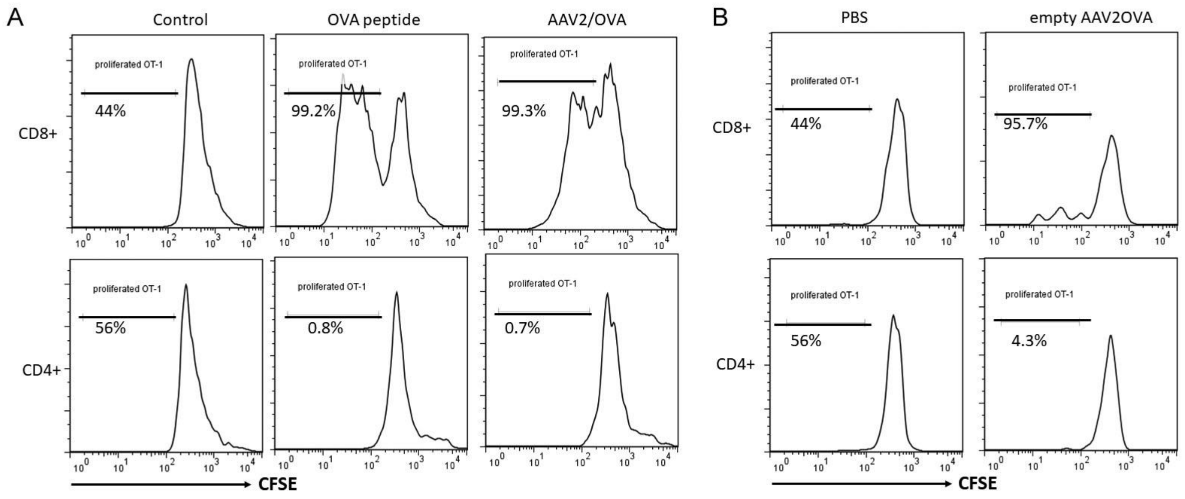

Figure 1. The proliferation of CD4+ and CD8+ OT-1 cells in the presence of OVA SIINFEKL antigen *in vitro* and *in vivo*. (A) HEK293/h2kb cells were incubated with OVA SIINFEKL peptides or AAV2OVA vectors overnight, CFSE labeled OT-1 spleen cells were added for 3 days. CD4+ or CD8+ CFSE labeled OT-1 cells in proliferation population were detected by flow cytometry. (B). $1X10^{11}$ empty AAV2OVA particles were administered into C57BL mice. One day later, CFSE labeled OT-1 spleen cells were infused. At day 7 post OT-1 cell transfusion, the proliferation of CD4+ or CD8+ CFSE labeled OT-1 cells was analyzed by flow cytometry.
